# Adaptive Control for Gravitational Wave Detection Formation Considering Time-Varying Communication Delays

**DOI:** 10.3390/s23063003

**Published:** 2023-03-10

**Authors:** Yu Zhang, Yuan Liu, Juzheng Zhang, Zhenkun Lu, Jikun Yang

**Affiliations:** 1 MOE Key Laboratory of TianQin Mission, TianQin Research Center for Gravitational Physics & School of Physics and Astronomy, Frontiers Science Center for TianQin, CNSA Research Center for Gravitational Waves, Sun Yat-Sen University (Zhuhai Campus), Zhuhai 519082, China; 2 School of Aeronautics and Astronautics, Sun Yat-Sen University, Shenzhen 518107, China

**Keywords:** distributed coordinated control, communication delays, adaptive control, dual quaternions

## Abstract

A distributed six-degree-of-freedom (6-DOF) cooperative control for multiple spacecraft formation is investigated considering parametric uncertainties, external disturbances, and time-varying communication delays. Unit dual quaternions are used to describe the kinematics and dynamics models of the 6-DOF relative motion of the spacecraft. A distributed coordinated controller based on dual quaternions with time-varying communication delays is proposed. The unknown mass and inertia, as well as unknown disturbances, are then taken into account. An adaptive coordinated control law is developed by combining the coordinated control algorithm with an adaptive algorithm to compensate for parametric uncertainties and external disturbances. The Lyapunov method is used to prove that the tracking errors converge globally asymptotically. Numerical simulations show that the proposed method can realize cooperative control of attitude and orbit for the multi-spacecraft formation.

## 1. Introduction

Einstein’s theory of general relativity was further proven when gravitational waves were first directly detected in LIGO observatories on 14 September 2015 [1]. Space gravitational wave detection has attracted more attention in detecting low-frequency gravitational wave signals. The United States has proposed the laser interferometric space antenna (LISA) [2], and China proposed the “Tianqin Project” [3] and the “Taiji Project” [4].

The space laser interferometer gravitational wave detector consists of three spacecraft, forming an equilateral triangle configuration with a scale of 100,000 km to 1 million km. By adjusting the attitude of the spacecraft, a laser link is established between the two spacecraft to detect gravitational waves. The space gravitational wave detection program currently has a relatively high requirement for configuration stability. However, the orbit injection error and perturbation will lead to the deviation between the actual orbit and the nominal orbit of the spacecraft, which will lead to the destruction of the equilateral triangle configuration and the destruction of the laser link, seriously affecting the implementation of scientific missions. At this time, the scientific observation of gravitational waves needs to be suspended for spacecraft formation configuration reconstruction and attitude adjustment. As is known, actuator configuration leads to dynamic coupling between rotation and translation [5]. In order to achieve high control accuracy of the system, the translation and the rotation of the spacecraft should be simultaneously taken into account. In recent years, Lie group SE(3) [6,7,8] and dual quaternions [9,10,11] have been the most popular methods to describe the coupling motion of rigid bodies. A 4 × 4 homogeneous transformation matrix is utilized when modeling rigid bodies on SE(3), while the model is described more compactly by dual quaternions, which have only eight parameters, and the dual quaternions’ multiplications have lower computational cost than homogeneous transformation matrix multiplications [12]. Therefore, this paper uses dual quaternion as a tool to design an attitude-orbit coupling coordination controller for space gravitational wave detection formation.

There are roughly five methods of multi-spacecraft coordination reported in the literature: the leader-following method [13,14,15], the behavior-based method [16], the virtual structure method [17], the artificial potential function method [18], and algebraic graph method [19]. The control laws in the previous works require precise inertial parameters of the spacecraft. However, obtaining accurate inertial parameters on fuel consumption during several years of gravitational wave detection missions is challenging. In addition, spacecraft working in the deep space environment are subject to unknown disturbances, including environmental and non-environmental forces and torques. Consequently, it is essential to design a cooperative control algorithm subject to parametric uncertainties and external disturbances for gravitational wave detection missions.

Adaptive control technology as an effective method to deal with parametric uncertainties and external disturbances has been widely used [20,21,22,23]. Wang et al. [24] proposed a robust tracking control of unknown models to deal with the problem of model uncertainties. In Ref. [25], a new adaptive nonsingular fast terminal sliding mode surface was developed for the attitude synchronization and tracking control of multiple spacecraft formation systems. Xing et al. [26] used a fuzzy logic system (FLS) to approximate the disturbance. Lin et al. [27] designed an adaptive fast integrating terminal sliding mode control law, which was robust to parameter uncertainties and external disturbances. The literature mentioned above mainly investigates the problem of attitude synchronization control for multiple spacecraft. Some scholars described spacecraft attitude and orbit motion in the dual quaternion framework and combined adaptive control and sliding mode control to deal with parameter uncertainties and external disturbances in spacecraft tracking control [9,28,29,30]. An adaptive tracking controller was designed in Ref. [10] for satellite proximity operations, which needs no information about the mass and inertia of the chaser spacecraft. On this basis, Gui et al. [11] improved the adaptive control law to reduce the control energy consumption. In Ref. [31], the problem of distributed finite time 6-DOF synchronization control for multiple spacecraft in the presence of external disturbances and parameter uncertainty was investigated, and 6-DOF coupled motion model was the Euler–Lagrange form. Nevertheless, to the best of our knowledge, there is little research on the dual quaternions-based adaptive coordinated controller design for multiple spacecraft.

The communication delay caused by the distance between neighboring spacecraft is another issue that deserves special attention. The current research on spacecraft formation control with communication delay mainly focuses on attitude coordination control [32,33,34]. In [7], the decentralized leaderless spacecraft consensus was studied considering a constant time delay between two spacecraft. Zhang et al. [8] proposed a nonsingular fast terminal sliding mode scheme to solve the consensus control problem of spacecraft formation flying in the presence of parametric uncertainties, external disturbances, and communication delays. Note that the relative position and attitude in the above works are represented on the Lie group SE(3). However, few studies in the literature discuss the attitude and orbit coupling coordinated control of multiple spacecraft considering parametric uncertainties, external disturbances, and time-varying communication delays in the framework of dual quaternions.

Inspired by this motivation, this paper mainly focuses on discussing the 6-DOF coordinated control problem for multiple spacecraft based on dual quaternions with consideration of parametric uncertainties, external disturbance, and time-varying communication delays. The main contributions in this paper can be summarized as follows:

(1) Dual quaternion is employed to describe the 6-DOF relative motion of the spacecraft. The gravitational force and torque, the perturbations due to the Earth’s $J_{2}$ oblateness, the solar pressure perturbation, and the constant external disturbances are considered;
(2) In the absence of modeling uncertainties and external disturbances, time-delay terms are added to the controller, which guarantees that the controller is effective to solve the cooperative control problem with communication delays;
(3) In the presence of modeling uncertainties and external disturbances, the cooperation controller with communication delays is developed into an adaptive controller, which can estimate the unknown parameters and external disturbances.

The rest of the paper is organized as follows: In Section 2, quaternions and dual quaternions are introduced. Then, a dual quaternion-based 6-DOF relative motion model is derived. Section 3 presents the proposed control laws and stability analysis. Finally, the simulation results verify the effectiveness of the proposed method in Section 4 followed by conclusions in Section 5.

## 2. Material Background and Relative Coupled Dynamics

### 2.1. Quaternions and Dual Quaternions

A quaternion is defined as $q=[\xi ,\bar{\eta }]$, where ξ∈R and $\bar{\eta }\in R^{3}$ are the scalar and vector part of the quaternion, respectively. The set of quaternions is defined as $H=\{q:q=\left(\xi ,\bar{\eta }\right)\}$. Let $H_{v}=\left\{q\in H:\xi =0\right\}$ and $H_{s}=\left\{q\in H:\bar{\eta }=0\right\}$ denote the set of vector quaternions and scalar quaternions, respectively. Given two quaternions $q_{1}=\left(\xi_{1},{\bar{q}}_{1}\right)$ and $q_{2}=\left(\xi_{2},{\bar{q}}_{2}\right)$ in H. The addition, multiplication, conjugation, dot product, and cross product are defined, respectively, by

(1) $$\begin{array}{ccc} q_{1}+q_{2} & = & (\xi_{1}+\xi_{2},{\bar{q}}_{1}+{\bar{q}}_{2})\in H \end{array}$$

(2) $$\begin{array}{ccc} q_{1}⊗q_{2} & = & (\xi_{1}\xi_{2}-{\bar{q}}_{1}\cdot {\bar{q}}_{2},\xi_{1}{\bar{q}}_{2}+\xi_{2}{\bar{q}}_{1}+{\bar{q}}_{1}\times {\bar{q}}_{2})\in H \end{array}$$

(3) $$\begin{array}{ccc} q^{*} & = & (\xi ,-\bar{q})\in H \end{array}$$

(4) $$\begin{array}{ccc} q_{1}\cdot q_{2} & = & \left(\xi_{1}\xi_{2}+{\bar{q}}_{1}\cdot {\bar{q}}_{2},\bar{0}\right)\in H_{s} \end{array}$$

(5) $$\begin{array}{ccc} q_{1}\times q_{2} & = & \left(0,\xi_{1}{\bar{q}}_{2}+\xi_{2}{\bar{q}}_{1}+{\bar{q}}_{1}\times {\bar{q}}_{2}\right)\in H_{v} \end{array}$$

A dual quaternion is defined as $\hat{q}=q_{r}+\varepsilon q_{d}$, where $q_{r}\in H$ and $q_{d}\in H$ are the real and dual parts, respectively. ε is the dual unit that satisfies the property $\varepsilon^{2}=0$ but ε≠0. The set of dual quaternions, dual vectors, and dual scalar quaternions are defined as $\mathrm{DQ}=\{\hat{q}:\hat{q}=q_{r}+\varepsilon q_{d}:q_{r},q_{d}\in H\}$, ${\mathrm{DQ}}_{v}=\left\{\hat{q}:\hat{q}=q_{r}+\varepsilon q_{d}:q_{r},q_{d}\in H_{v}\right\}$, ${\mathrm{DQ}}_{s}=\left\{\hat{q}:\hat{q}=q_{r}+\varepsilon q_{d}:q_{r},q_{d}\in H_{s}\right\}$, respectively. The set of dual scalar quaternions with zero dual part is denoted by ${\mathrm{DQ}}_{r}=\left\{\hat{q}:\hat{q}=q+\varepsilon 0:q\in H_{s}\right\}$.

Given two dual quaternions ${\hat{q}}_{1}=q_{1r}+\varepsilon q_{1d}$ and ${\hat{q}}_{2}=q_{2r}+\varepsilon q_{2d}$ in DQ with $q_{1r}$, $q_{1d}$, $q_{2r}$, and $q_{2d}$ in H. The addition, multiplication, conjugation, dot product, and cross product are defined, respectively, by

(6) $$\begin{array}{ccc} {\hat{q}}_{1}+{\hat{q}}_{2} & = & \left(q_{1r}+q_{2r}\right)+\varepsilon \left(q_{1d}+q_{2d}\right)\in \mathrm{DQ} \end{array}$$

(7) $$\begin{array}{ccc} {\hat{q}}_{1}⊗{\hat{q}}_{2} & = & \left(q_{1r}⊗q_{2r}\right)+\varepsilon \left(q_{1r}⊗q_{2d}+q_{1d}⊗q_{2r}\right)\in \mathrm{DQ} \end{array}$$

(8) $$\begin{array}{ccc} {\hat{q}}^{*} & = & {q_{r}}^{*}+\varepsilon {q_{d}}^{*}\in \mathrm{DQ} \end{array}$$

(9) $$\begin{array}{ccc} {\hat{q}}_{1}\cdot {\hat{q}}_{2} & = & q_{1r}\cdot q_{2r}+\varepsilon \left(q_{1d}\cdot q_{2r}+q_{1r}\cdot q_{2d}\right)\in {\mathrm{DQ}}_{s} \end{array}$$

(10) $$\begin{array}{ccc} {\hat{q}}_{1}\times {\hat{q}}_{2} & = & q_{1r}\times q_{2r}+\varepsilon \left(q_{1d}\times q_{2r}+q_{1r}\times q_{2d}\right)\in {\mathrm{DQ}}_{v} \end{array}$$

The swap product of a dual quaternion is ${\hat{q}}^{s}=q_{d}+\varepsilon q_{r}\in \mathrm{DQ}$. The ⊙ product of a dual quaternion is $\hat{c}⊙\hat{q}=\left(c_{r}+\varepsilon c_{d}\right)⊙\left(q_{r}+\varepsilon q_{d}\right)=c_{r}q_{r}+\varepsilon c_{d}q_{d},\hat{q}\in \mathrm{DQ}$. The circle product of two dual quaternions is ${\hat{q}}_{1}\circ {\hat{q}}_{2}=q_{1r}\cdot q_{2r}+q_{1d}\cdot q_{2d},{\hat{q}}_{1},{\hat{q}}_{2}\in {\mathrm{DQ}}_{v}$.

The following properties with the above definitions can be shown:

(11) $$\hat{a}\circ \left(\hat{b}⊗\hat{c}\right)={\hat{b}}^{s}\circ \left({\hat{a}}^{s}⊗{\hat{c}}^{*}\right)={\hat{c}}^{s}\circ \left({\hat{b}}^{*}⊗{\hat{a}}^{s}\right)\in R,\hat{a},\hat{b},\hat{c}\in \mathrm{DQ}$$

(12) $$\hat{a}\circ \left(\hat{b}\times \hat{c}\right)={\hat{b}}^{s}\circ \left(\hat{c}\times {\hat{a}}^{s}\right)={\hat{c}}^{s}\circ \left({\hat{a}}^{s}\times \hat{b}\right)\in R,\hat{a},\hat{b},\hat{c}\in {\mathrm{DQ}}_{v}$$

(13) $${\hat{a}}^{s}\circ {\hat{b}}^{s}=\hat{a}\circ \hat{b},\hat{a},\hat{b}\in \mathrm{DQ}$$

(14) $$\left|\right|\hat{a}{\left|\right|}^{2}=\hat{a}\circ \hat{a},\hat{a}\in {\mathrm{DQ}}_{r}$$

### 2.2. Equations of 6-DOF Relative Motion Based on Dual Quaternions

The 6-DOF relative motion model of spacecraft based on dual quaternions is established in this subsection. Let $F_{I}$ represent the Earth-centered-inertial frame with the origin at the center of the Earth. The body-fixed coordinate system $F_{i}$ (*i* means the *i*-th spacecraft) is defined with the origin at the center of mass.

The kinematics equation of the *i*-th spacecraft is given by [35]

(15) $${\dot{\hat{q}}}_{i}=\frac{1}{2}{\hat{q}}_{i}⊗{\hat{\omega }}_{i}^{i}$$

where ${\hat{q}}_{i}=q_{i}+\varepsilon \frac{1}{2}q_{i}⊗r_{i}^{i}$. $r_{i}^{i}=\left[0,{\bar{r}}_{i}^{i}\right]$, ${\bar{r}}_{i}^{i}\in R^{3}$ is the translation vector from the origin of the frame $F_{I}$ to the origin of the frame $F_{i}$ expressed in the frame $F_{i}$. $q_{i}$ denotes the orientation of the frame $F_{i}$ relative to the frame $F_{I}$ in terms of unit quaternion. ${\hat{\omega }}_{i}^{i}$ denotes the dual velocity of the *i*-th spacecraft, given in the body-fixed frame $F_{i}$, which is defined as

(16) $${\hat{\omega }}_{i}^{i}=\omega_{i}^{i}+\varepsilon \left({\dot{r}}_{i}^{i}+\omega_{i}^{i}\times r_{i}^{i}\right)$$

where $\omega_{i}^{i}=\left[0,{\bar{\omega }}_{i}^{i}\right]$, ${\bar{\omega }}_{i}^{i}\in R^{3}$ is the angular velocity of the *i*-th spacecraft expressed in the frame $F_{i}$. ${\hat{M}}_{i}$ is the dual inertia matrix, which is defined as [11]

(17) $$\begin{array}{cc} {\hat{M}}_{i}\; & =m_{i}\frac{d}{d\varepsilon }I_{3}+\varepsilon J_{i}\\ \; & =\left[\begin{array}{ccc} m_{i}\frac{d}{d\varepsilon }+\varepsilon J_{i11} & \varepsilon J_{i12} & \varepsilon J_{i13}\\ \varepsilon J_{i21} & m_{i}\frac{d}{d\varepsilon }+\varepsilon J_{i22} & \varepsilon J_{i23}\\ \varepsilon J_{i31} & \varepsilon J_{i32} & m_{i}\frac{d}{d\varepsilon }+\varepsilon J_{i33} \end{array}\right] \end{array}$$

where $m_{i}$ and $J_{i}$ are the mass and inertia matrix of the *i*-th spacecraft. The operator $\frac{d}{d\varepsilon }$ is defined by $\frac{d}{d\varepsilon }\hat{a}=\frac{d}{d\varepsilon }\left(a_{r}+\varepsilon a_{d}\right)=a_{d}$ and ${\left(\frac{d}{d\varepsilon }\right)}^{2}=0$. $I_{3}$ is the identity in dimension 3. The inverse of ${\hat{M}}_{i}$ is defined as ${\hat{M}}_{i}^{-1}=J_{i}^{-1}\frac{d}{d\varepsilon }+\varepsilon \frac{1}{m_{i}}I_{3}$ [36].

The dual quaternion representation of the *i*-th spacecraft dynamics equation is given by [35]

(18) $${\hat{M}}_{i}{\dot{\hat{\omega }}}_{i}^{i}={\hat{F}}_{i}^{i}-{\hat{\omega }}_{i}^{i}\times {\hat{M}}_{i}{\hat{\omega }}_{i}^{i}$$

For the case of gravitational wave detection in Earth orbit, the total dual force acting on the spacecraft will be decomposed as follows:

(19) $${\hat{F}}_{i}^{i}={\hat{f}}_{gi}^{i}+{\hat{f}}_{srpi}^{i}+{\hat{f}}_{J_{2}i}^{i}+{\hat{f}}_{▽gi}^{i}+{\hat{f}}_{di}^{i}+{\hat{f}}_{ui}^{i}$$

where ${\hat{f}}_{gi}^{i}={\hat{M}}_{i}{\hat{a}}_{gi}^{i}$, ${\hat{a}}_{gi}^{i}=0+\varepsilon \left[0,{\bar{a}}_{gi}^{i}\right]$, ${\bar{a}}_{gi}^{i}$ is the gravitational acceleration, including the Earth, Moon, and Sun, given by

(20) $${\bar{a}}_{gi}^{i}=-\frac{\mu_{e}{\bar{r}}_{i}^{i}}{∥{\bar{r}}_{i}^{i}{∥}^{3}}-\mu_{m}\left(\frac{{\bar{r}}_{i}^{i}-{\bar{r}}_{m}^{i}}{∥{\bar{r}}_{i}^{i}-{\bar{r}}_{m}^{i}{∥}^{3}}+\frac{{\bar{r}}_{m}^{i}}{∥{\bar{r}}_{m}^{i}{∥}^{3}}\right)-\mu_{s}\left(\frac{{\bar{r}}_{i}^{i}-{\bar{r}}_{s}^{i}}{∥{\bar{r}}_{i}^{i}-{\bar{r}}_{s}^{i}{∥}^{3}}+\frac{{\bar{r}}_{s}^{i}}{∥{\bar{r}}_{s}^{i}{∥}^{3}}\right)$$

where $\mu_{e}=398,600.44190$${\mathrm{km}}^{3}/s^{2}$, $\mu_{m}=4902.800076$${\mathrm{km}}^{3}/s^{2}$ and $\mu_{s}=132,712,440,040.94400$ ${\mathrm{km}}^{3}/s^{2}$ are the gravitational parameter of the Earth, Moon, and Sun, respectively. ${\bar{r}}_{m}^{i}$ and ${\bar{r}}_{s}^{i}$ denote the position vector of the Moon and Sun relative to the Earth expressed in $F_{i}$. ${\hat{f}}_{srpi}^{i}={\hat{M}}_{i}{\hat{a}}_{srpi}^{i}$, ${\hat{a}}_{srpi}^{i}=0+\varepsilon \left[0,{\bar{a}}_{srpi}^{i}\right]$, the acceleration ${\bar{a}}_{srpi}^{i}$ caused by solar radiation pressure can be approximately expressed as

(21) $${\bar{a}}_{srpi}^{i}=-P_{⊙}\frac{A}{m_{i}}\frac{r_{⊙}}{r_{⊙}^{3}}AU^{2}\left(1+\epsilon \right)$$

where $P_{⊙}=4.56\times 10^{-6}$${\mathrm{Nm}}^{2}$ is the solar radiation pressure at 1AU (Astronomical Unit), *A* the occulted segment of the Sun, $r_{⊙}$ the position vector from the Sun to the spacecraft, ϵ the reflectivity of the surface. ${\hat{f}}_{J_{2}i}^{i}={\hat{M}}_{i}{\hat{a}}_{J_{2}i}^{i}$, ${\hat{a}}_{J_{2}i}^{i}=0+\varepsilon a_{J_{2}i}^{i}$, $a_{J_{2}i}^{i}=\left[0,{\bar{a}}_{J_{2}i}^{i}\right]=q_{i}^{*}⊗a_{J_{2}i}^{I}⊗q_{i}$, and $a_{J_{2}i}^{I}=\left[0,{\bar{a}}_{J_{2}i}^{I}\right]$, ${\bar{a}}_{J_{2}i}^{I}$ is the perturbing acceleration due to Earth’s oblateness given by

(22) $${\bar{a}}_{J_{2}i}^{I}=-\frac{3}{2}\frac{\mu_{e}J_{2}R_{e}^{2}}{∥{\bar{r}}_{i}^{I}{∥}^{5}}\left(D-5{\left(\frac{r_{i}^{z}}{∥{\bar{r}}_{i}^{I}∥}\right)}^{2}I_{3}\right){\bar{r}}_{i}^{I}$$

where $R_{e}=6378.137$ km is the Earth’s mean equatorial radius, $J_{2}=0.0010826267$, D= diag{1,1,3}; ${\bar{r}}_{i}^{I}={\left[r_{i}^{x},r_{i}^{y},r_{i}^{z}\right]}^{T}$ represents the coordinates of ${\bar{r}}_{i}$ expressed in the inertial coordinate system. The ${\hat{f}}_{▽gi}^{i}$ is the dual force due to the gravity-gradient torque, defined as

(23) $${\hat{f}}_{▽gi}^{i}=3\mu_{e}\frac{{\hat{r}}_{i}^{i}\times {\hat{M}}_{i}{\hat{r}}_{i}^{i}}{∥r_{i}^{i}{∥}^{5}}$$

where ${\hat{r}}_{i}^{i}=r_{i}^{i}+\varepsilon 0$.${\hat{f}}_{di}^{i}=f_{di}^{i}+\varepsilon \tau_{di}^{i}$ and ${\hat{f}}_{ui}^{i}=f_{ui}^{i}+\varepsilon \tau_{ui}^{i}$ are the dual disturbance force and the dual control force, with the disturbance force $f_{di}^{i}$, the disturbance torque $\tau_{di}^{i}$, the control force $f_{ui}^{i}$ and the control torque $\tau_{ui}^{i}$ given in the body-fixed frame $F_{i}$, respectively.

Under the dual quaternion algebra, the motion between the body-fixed frame and its desired frame can be expressed in the $F_{i}$ as the relative dual quaternion described by

(24) $${\hat{q}}_{ei}={\hat{q}}_{di}^{*}⊗{\hat{q}}_{i}=q_{ei}+\varepsilon \frac{1}{2}q_{ei}⊗r_{ei}^{i}$$

where ${\hat{q}}_{di}^{*}$ is the conjugate of ${\hat{q}}_{di}$. ${\hat{q}}_{di}$ denotes the dual quaternion of the frame $F_{i}$ relative to the frame $F_{I}$. $q_{ei}$ denotes the orientation of the frame $F_{i}$ relative to the frame $F_{di}$ in terms of unit quaternion. $r_{ei}^{i}$ is the relative position between the *i*-th spacecraft and its desired position, given in the $F_{i}$. The relative kinematic and dynamic equations are given by

(25) $${\dot{\hat{q}}}_{ei}=\frac{1}{2}{\hat{q}}_{ei}⊗{\hat{\omega }}_{ei}^{i}$$

(26) $$\begin{array}{cc} {\hat{M}}_{i}{\dot{\hat{\omega }}}_{ei}^{i}\; & ={\hat{F}}_{i}^{i}-{\hat{\omega }}_{i}^{i}\times {\hat{M}}_{i}{\hat{\omega }}_{i}^{i}+{\hat{M}}_{i}\left({\hat{\omega }}_{ei}^{i}\times {\hat{\omega }}_{di}^{i}\right)-{\hat{M}}_{i}\left({\hat{q}}_{ei}^{*}⊗{\dot{\hat{\omega }}}_{di}^{di}⊗{\hat{q}}_{ei}\right) \end{array}$$

where ${\hat{\omega }}_{ei}^{i}$ is the dual velocity between the $F_{i}$ and $F_{di}$, expressed in the $F_{i}$. The kinematics and dynamic models of the desired *i*-th spacecraft are similar to the *i*-th spacecraft, which corresponds to (15), (16), and (18), where the notations ’${•}_{i}$’ and ’${•}_{i}^{i}$’ are replaced by ’${•}_{di}$’ and ’${•}_{di}^{di}$’. The total dual force applied to the desired *i*-th spacecraft is independent of the dual disturbance and dual control force, i.e., ${\hat{F}}_{di}^{di}={\hat{f}}_{gdi}^{di}+{\hat{f}}_{J_{2}di}^{di}+{\hat{f}}_{▽gdi}^{di}$.

### 2.3. Control Objective

In this paper, (${\hat{q}}_{di}\left(t\right),{\hat{\omega }}_{di}^{di}\left(t\right)$) denotes the desired state information of the *i*-th spacecraft to meet the requirements of gravitational wave detection. (${\hat{q}}_{i}\left(t\right),{\hat{\omega }}_{i}^{i}\left(t\right)$) denotes the actual state information of the *i*-th spacecraft. The objective of this paper is to design an adaptive cooperation control scheme based on dual quaternions such that the state (${\hat{q}}_{i}\left(t\right),{\hat{\omega }}_{i}^{i}\left(t\right)$) can track its desired state (${\hat{q}}_{di}\left(t\right),{\hat{\omega }}_{di}^{di}\left(t\right)$) in the presence of parametric uncertainties, external disturbances, and time-varying communication delays. In other words, the error state (${\hat{q}}_{ei}\left(t\right),{\hat{\omega }}_{ei}^{i}\left(t\right)$) can converge to an arbitrarily small neighborhood of the origin. That is, when t→∞,

(27) $$\begin{array}{cc} {\hat{q}}_{ei}\left(t\right) & \to \pm \hat{1}\\ {\hat{\omega }}_{ei}^{i}\left(t\right) & \to \hat{0} \end{array}$$

where $\hat{1}=1+\varepsilon 0\in \mathrm{DQ}$, $\hat{0}=0+\varepsilon 0\in \mathrm{DQ}$, $1=(1,\bar{0})\in H$ and $0=(0,\bar{0})\in H$, respectively.

## 3. Control Law Design

In this section, a distributed coordinated formation control law is designed to solve the 6-DOF coordination control problem with the time-varying communication delays. Then, an adaptive controller is developed to provide the estimations of the parametric uncertainties and external disturbances.

### 3.1. 6-DOF Coordinated Control Law with Communication Delays

This subsection considers the time-varying communication delays between spacecraft, regardless of parametric uncertainties and external disturbances. An auxiliary state ${\hat{s}}_{i}$ is first defined as

(28) $${\hat{s}}_{i}={\hat{\omega }}_{ei}^{i}+\hat{c}⊙{\hat{p}}_{ei}^{i}$$

where $\hat{c}=c_{r}+\varepsilon c_{d}$ with $c_{r}$ and $c_{d}$ are all positive constants. ${\hat{p}}_{ei}^{i}$ is defined as ${\hat{p}}_{ei}^{i}=vec\left(q_{ei}\right)+\varepsilon \frac{1}{2}r_{ei}^{i}$, $vec(q_{ei})$ is the vector part of $q_{ei}$. When the system dynamics are exactly known, a distributed coordinated controller with communication delays is proposed in the following form:

(29) $$\begin{array}{cc} {\hat{f}}_{ui}^{i}= & -{\hat{k}}_{1}⊙{\left({\hat{s}}_{i}\right)}^{s}-{\hat{k}}_{2}⊙\sum_{j=1}^{n}a_{ij}{\left({\hat{s}}_{i}-{\hat{s}}_{j}\left(t-T_{ij}\right)\right)}^{s}+{\hat{M}}_{i}Y_{i}-{\hat{f}}_{di}^{i} \end{array}$$

(30) $$\begin{array}{cc} Y_{i}= & {\hat{M}}_{i}^{-1}\left({\hat{\omega }}_{i}^{i}\times {\hat{M}}_{i}{\hat{\omega }}_{i}^{i}\right)-{\hat{\omega }}_{ei}^{i}\times {\hat{\omega }}_{di}^{i}+{\hat{q}}_{ei}^{*}⊗{\dot{\hat{\omega }}}_{di}^{di}⊗{\hat{q}}_{ei}-{\hat{a}}_{gi}^{i}\\ \; & -{\hat{a}}_{srpi}^{i}-{\hat{a}}_{J_{2}i}^{i}-{\hat{a}}_{▽gi}^{i}-\hat{c}⊙{\dot{\hat{p}}}_{ei}^{i} \end{array}$$

where ${\hat{k}}_{1}=k_{1r}+\varepsilon k_{1d}$ and ${\hat{k}}_{2}=k_{2r}+\varepsilon k_{2d}$, with $k_{1r},k_{1d},k_{2r},k_{2d}$ all being positive constants. It assumed that the communication topology between the *i*-th and the *j*-th spacecraft is undirected. Therefore, $a_{ij}=1,i\neq j$. Otherwise, $a_{ij}=0$. $T_{ij}$ is the time-varying communication delay from the *j*-th to *i*-th spacecraft.

**Theorem** **1.** *Consider the relative kinematic and dynamic equations given by Equations (25) and (26), and the undirected communication graph is connected. If the time derivative of $T_{ij}$ satisfies ${\dot{T}}_{ij}\leq 0$, the distributed coordinated formation control law in Equation (29) can ensure $\underset{t\to \infty }{lim}\left({\hat{q}}_{ei},{\hat{\omega }}_{ei}^{i}\right)\left(t\right)=\left(\pm \hat{1},\hat{0}\right)$ for all initial conditions.*

**Proof** **of** **Theorem** **1.** Consider the Lyapunov function candidate $V_{1}=V_{1a}+V_{1b}$, where

(31) $$\begin{array}{cc} V_{1a} & =\frac{1}{2}\sum_{i=1}^{n}{\left({\hat{s}}_{i}\right)}^{s}\circ \left({\hat{M}}_{i}{\hat{s}}_{i}\right) \end{array}$$

(32) $$\begin{array}{cc} V_{1b} & =\frac{1}{2}{\hat{k}}_{2}⊙\sum_{i=1}^{n}\sum_{j=1}^{n}a_{ij}\int_{t-T_{ij}}^{t}{\hat{s}}_{j}\left(\tau \right)\circ {\hat{s}}_{j}\left(\tau \right)d\tau \end{array}$$

It can be verified that $V_{1}\geq 0$ for all ${\hat{s}}_{i}$ and $V_{1}=0$ if and only if ${\hat{s}}_{i}=\hat{0}$.

Taking the time derivative of $V_{1a}$ and $V_{1b}$ along the trajectories of the formation system (25) and (26), we can obtain

(33) $$\begin{array}{cc} {\dot{V}}_{1a} & =\sum_{i=1}^{n}{\left({\hat{s}}_{i}\right)}^{s}\circ \left({\hat{M}}_{i}{\dot{\hat{s}}}_{i}\right) \end{array}$$

(34) $$\begin{array}{cc} {\dot{V}}_{1b}= & \frac{1}{2}{\hat{k}}_{2}⊙\sum_{i=1}^{n}\sum_{j=1}^{n}a_{ij}\left({\hat{s}}_{j}\left(t\right)\circ {\hat{s}}_{j}\left(t\right)-\left(1-{\dot{T}}_{ij}\right){\hat{s}}_{j}\left(t-T_{ij}\right)\circ {\hat{s}}_{j}\left(t-T_{ij}\right)\right) \end{array}$$

By taking a derivative of (28), substituting it into (33) yields

(35) $$\begin{array}{cc} {\dot{V}}_{1a} & =\sum_{i=1}^{n}{\left({\hat{s}}_{i}\right)}^{s}\circ {\hat{M}}_{i}\left({\dot{\hat{\omega }}}_{ei}^{i}+\hat{c}⊙{\dot{\hat{p}}}_{ei}^{i}\right)\\ \; & =\sum_{i=1}^{n}{\left({\hat{s}}_{i}\right)}^{s}\circ \left(-{\hat{k}}_{1}⊙{\left({\hat{s}}_{i}\right)}^{s}-{\hat{k}}_{2}⊙\sum_{j=1}^{n}a_{ij}{\left({\hat{s}}_{i}-{\hat{s}}_{j}\left(t-T_{ij}\right)\right)}^{s}\right) \end{array}$$

Then,

(36) $$\begin{array}{cc} {\dot{V}}_{1}= & {\dot{V}}_{1a}+{\dot{V}}_{1b}\\ = & \sum_{i=1}^{n}{\left({\hat{s}}_{i}\right)}^{s}\circ \left(-{\hat{k}}_{1}⊙{\left({\hat{s}}_{i}\right)}^{s}-{\hat{k}}_{2}⊙\sum_{j=1}^{n}a_{ij}{\left({\hat{s}}_{i}-{\hat{s}}_{j}\left(t-T_{ij}\right)\right)}^{s}\right)\\ \; & +\frac{1}{2}{\hat{k}}_{2}⊙\sum_{i=1}^{n}\sum_{j=1}^{n}a_{ij}\left({\left({\hat{s}}_{j}\right)}^{s}\circ {\left({\hat{s}}_{j}\right)}^{s}\right)\\ \; & -\frac{\left(1-{\dot{T}}_{ij}\right)}{2}{\hat{k}}_{2}⊙\sum_{i=1}^{n}\sum_{j=1}^{n}a_{ij}\left({\left({\hat{s}}_{j}\left(t-T_{ij}\right)\right)}^{s}\circ {\left({\hat{s}}_{j}\left(t-T_{ij}\right)\right)}^{s}\right) \end{array}$$

Note that the undirected topology is balanced, meaning that $\sum_{j=1}^{n}a_{ij}=\sum_{j=1}^{n}a_{ji}$ for i=1,…n; then, it follows that

(37) $$\begin{array}{c} \sum_{i=1}^{n}\sum_{j=1}^{n}a_{ij}{\hat{s}}_{i}=\sum_{j=1}^{n}\sum_{i=1}^{n}a_{ji}{\hat{s}}_{i}=\sum_{i=1}^{n}\sum_{j=1}^{n}a_{ij}{\hat{s}}_{j} \end{array}$$

Then,

(38) $$\begin{array}{cc} {\dot{V}}_{1}= & -{\hat{k}}_{1}⊙\sum_{i=1}^{n}{\left({\hat{s}}_{i}\right)}^{s}\circ {\left({\hat{s}}_{i}\right)}^{s}+{\dot{T}}_{ij}{\hat{k}}_{2}⊙\sum_{i=1}^{n}\sum_{j=1}^{n}a_{ij}{\left({\hat{s}}_{j}\left(t-T_{ij}\right)\right)}^{s}\circ {\left({\hat{s}}_{j}\left(t-T_{ij}\right)\right)}^{s}\\ \; & -{\hat{k}}_{2}⊙\sum_{i=1}^{n}\sum_{j=1}^{n}a_{ij}{\left({\hat{s}}_{i}-{\hat{s}}_{j}\left(t-T_{ij}\right)\right)}^{s}\circ {\left({\hat{s}}_{i}-{\hat{s}}_{j}\left(t-T_{ij}\right)\right)}^{s} \end{array}$$

Therefore, if ${\dot{T}}_{ij}<0$, it follows that ${\dot{V}}_{1}\left(t\right)\leq 0$. Since $V_{1}\left(t\right)\geq 0$ and ${\dot{V}}_{1}\left(t\right)\leq 0$, ${\hat{s}}_{i}$ is bounded. According to Equation (28), the boundedness of ${\hat{s}}_{i}$ means that ${\hat{p}}_{ei}^{i}$ and ${\hat{\omega }}_{ei}^{i}$ are bounded. In addition, the boundedness of ${\hat{p}}_{ei}^{i}$ and ${\hat{\omega }}_{ei}^{i}$ means that ${\hat{f}}_{ui}^{i}$ and thus ${\dot{\hat{\omega }}}_{ei}^{i}$ are bounded. Hence, ${\hat{\omega }}_{ei}^{i}$ and ${\dot{V}}_{1}$ are uniformly continuous. It follows from Barbalat’s lemma that $\underset{t\to \infty }{lim}{\dot{V}}_{1}\left(t\right)=0$ and thus $\underset{t\to \infty }{lim}{\dot{\hat{\omega }}}_{ei}^{i}\left(t\right)=\hat{0}$. Furthermore, since ${\ddot{\hat{\omega }}}_{ei}^{i}$ is bounded and thus ${\dot{\hat{\omega }}}_{ei}^{i}$ is uniformly continuous, it follows from Barbalat’s lemma that $\underset{t\to \infty }{lim}{\hat{\omega }}_{ei}^{i}\left(t\right)=\hat{0}$ and thus $\underset{t\to \infty }{lim}{\hat{p}}_{ei}^{i}\left(t\right)=\hat{0}$, which implies $\underset{t\to \infty }{lim}\left({\hat{q}}_{ei},{\hat{\omega }}_{ei}^{i}\right)\left(t\right)=\left(\pm \hat{1},\hat{0}\right)$. (Note that ${\hat{q}}_{ei}=\hat{1}$ and ${\hat{q}}_{ei}=-\hat{1}$ are the same pose.) Thus, the control objective is achieved. □

### 3.2. Adaptive 6-DOF Coordinated Control Law with Communication Delays

In this subsection, let us consider the delayed 6-DOF coordination control problem with model and disturbances uncertainties and propose an adaptive coordinated controller in the following form:

(39) $${\hat{f}}_{ui}^{i}=-{\hat{k}}_{1}⊙{\left({\hat{s}}_{i}\right)}^{s}-{\hat{k}}_{2}⊙\sum_{j=1}^{n}a_{ij}{\left({\hat{s}}_{i}-{\hat{s}}_{j}\left(t-T_{ij}\right)\right)}^{s}+{\tilde{M}}_{i}Y_{i}-{\tilde{f}}_{di}^{i}$$

(40) $$\begin{array}{cc} Y_{i}= & {\tilde{M}}_{i}^{-1}\left({\hat{\omega }}_{i}^{i}\times {\tilde{M}}_{i}{\hat{\omega }}_{i}^{i}\right)-{\hat{\omega }}_{ei}^{i}\times {\hat{\omega }}_{di}^{i}+{\hat{q}}_{ei}^{*}⊗{\dot{\hat{\omega }}}_{di}^{di}⊗{\hat{q}}_{ei}-{\hat{a}}_{gi}^{i}\\ \; & -{\hat{a}}_{srpi}^{i}-{\hat{a}}_{J_{2}i}^{i}-{\hat{a}}_{▽gi}^{i}-\hat{c}⊙{\dot{\hat{p}}}_{ei}^{i} \end{array}$$

where ${\tilde{M}}_{i}$ and ${\tilde{f}}_{di}^{i}$ are the estimation of ${\hat{M}}_{i}$ and ${\hat{f}}_{di}^{i}$, respectively. All gains are the same as the ones in the last subsection. To simplify notation, the following is introduced as

(41) $$\hat{a}\circ \left(\hat{M_{i}}\hat{b}\right)=h^{T}\left(\hat{a},\hat{b}\right)\Gamma \left(\hat{M_{i}}\right)$$

where $\hat{a}=a_{r}+\varepsilon a_{d}$, $\hat{b}=b_{r}+\varepsilon b_{d}$ are dual quaternions, with $a_{r}=\left[a_{r0},a_{r1},a_{r2},a_{r3}\right]$, $a_{d}=\left[a_{d0},a_{d1},a_{d2},a_{d3}\right]$, $b_{r}=\left[b_{r0},b_{r1},b_{r2},b_{r3}\right]$, $b_{d}=\left[b_{d0},b_{d1},b_{d2},b_{d3}\right]$. The function h is defined as $h\left(\hat{a},\hat{b}\right)=[a_{d1}b_{r1},a_{d2}b_{r1}+a_{d1}b_{r2},a_{d3}b_{r1}+a_{d1}b_{r3},a_{d2}b_{r2},a_{d3}b_{r2}+a_{d2}b_{r3},a_{d3}a_{r3},a_{r1}b_{d1}$$+a_{r2}b_{d2}+a_{r3}b_{d3}{]}^{T}$, and $\Gamma \left(\hat{M_{i}}\right)={\left[J_{i11},J_{i12},J_{i13},J_{i22},J_{i23},J_{i33},m_{i}\right]}^{T}$. The updating law for ${\tilde{M}}_{i}$ and ${\tilde{f}}_{di}^{i}$ can be designed as

(42) $$\begin{array}{cc} \frac{d}{dt}\Gamma \left({\tilde{M}}_{i}\right)= & W_{M}[h\left({\left({\hat{s}}_{i}\right)}^{s},{\hat{\omega }}_{ei}^{i}\times {\hat{\omega }}_{di}^{i}-{\hat{q}}_{ei}^{*}⊗{\dot{\hat{\omega }}}_{di}^{di}⊗{\hat{q}}_{ei}+{\hat{a}}_{gi}^{i}+{\hat{a}}_{srpi}^{i}+{\hat{a}}_{J_{2}i}^{i}+\hat{c}⊙{\dot{\hat{p}}}_{ei}^{i}\right)\\ \; & -h\left({\left({\hat{s}}_{i}\times {\hat{\omega }}_{i}^{i}\right)}^{s},{\hat{\omega }}_{i}^{i}\right)+h\left({\left({\hat{s}}_{i}\times \frac{3\mu_{e}{\hat{r}}_{i}^{i}}{∥r_{i}^{i}{∥}^{5}}\right)}^{s},{\hat{r}}_{i}^{i}\right)] \end{array}$$

(43) $$\begin{array}{c} \frac{d}{dt}{\tilde{f}}_{di}^{i}={\hat{W}}_{d}{\left({\hat{s}}_{i}\right)}^{s} \end{array}$$

where $W_{M}\in R^{7\times 7}$ is a positive definite matrix. ${\hat{W}}_{d}=W_{d2}\frac{d}{d\varepsilon }+\varepsilon W_{d1}$ with $W_{d1}$ and $W_{d2}\in R^{3\times 3}$ being positive definite matrices.

**Theorem** **2.** *Consider the relative kinematic and dynamic equations given by Equations (25) and (26), and the undirected communication graph is connected. If the time derivative of $T_{ij}$ satisfies ${\dot{T}}_{ij}\leq 0$, the distributed coordinated formation control law in Equations (39) and (40), with the adaptive law (42) and (43) can ensure $\underset{t\to \infty }{lim}\left({\hat{q}}_{ei},{\hat{\omega }}_{ei}^{i}\right)\left(t\right)=\left(\pm \hat{1},\hat{0}\right)$ for all initial conditions.*

**Proof** **of** **Theorem** **2.** The dual inertia matrix and dual disturbance force estimation errors are defined as $\Delta \hat{M_{i}}=\tilde{M_{i}}-\hat{M_{i}}$ and $\Delta {\hat{f}}_{di}^{i}={\tilde{f}}_{di}^{i}-{\hat{f}}_{di}^{i}$, respectively. Consider a Lyapunov function candidate as $V_{2}=V_{2a}+V_{2b}+V_{2c}$, where

(44) $$\begin{array}{cc} V_{2a} & =\frac{1}{2}\sum_{i=1}^{n}{\left({\hat{s}}_{i}\right)}^{s}\circ \left({\hat{M}}_{i}{\hat{s}}_{i}\right)\\ V_{2b} & =\frac{1}{2}{\hat{k}}_{2}⊙\sum_{i=1}^{n}\sum_{j=1}^{n}a_{ij}\int_{t-T_{ij}}^{t}{\left({\hat{s}}_{j}\left(\tau \right)\right)}^{s}\circ {\left({\hat{s}}_{j}\left(\tau \right)\right)}^{s}d\tau \\ V_{2c} & =\frac{1}{2}\sum_{i=1}^{n}\Gamma^{T}\left(\Delta \hat{M_{i}}\right)W_{M}^{-1}\Gamma \left(\Delta \hat{M_{i}}\right)+\frac{1}{2}\sum_{i=1}^{n}\Delta {\hat{f}}_{di}^{i}\circ \left({\hat{W}}_{d}^{-1}\Delta {\hat{f}}_{di}^{i}\right) \end{array}$$

$V_{2}$ is a valid candidate Lyapunov function since $V_{2}\geq 0$ for all ${\hat{s}}_{i}$, $\Gamma^{T}\left(\Delta \hat{M_{i}}\right)$ and $\Delta {\hat{f}}_{di}^{i}$; $V_{2}=0$ if and only if ${\hat{s}}_{i}=\hat{0}$, $\Gamma^{T}\left(\Delta \hat{M_{i}}\right)=0_{7\times 1}$ and $\Delta {\hat{f}}_{di}^{i}=\hat{0}$.

By taking the derivative of Formula (44), we can arrive at

(45) $$\begin{array}{cc} {\dot{V}}_{2a}= & \sum_{i=1}^{n}{\left({\hat{s}}_{i}\right)}^{s}\circ \left({\hat{M}}_{i}{\dot{\hat{s}}}_{i}\right)\\ = & \sum_{i=1}^{n}{\left({\hat{s}}_{i}\right)}^{s}\circ {\left(-{\hat{k}}_{1}⊙{\left({\hat{s}}_{i}\right)}^{s}-{\hat{k}}_{2}⊙\sum_{j=1}^{n}a_{ij}\left({\hat{s}}_{i}-{\hat{s}}_{j}(t-T_{ij}\right)\right)}^{s}\\ \; & -\sum_{i=1}^{n}\Gamma^{T}\left(\Delta \hat{M_{i}}\right)W_{M}^{-1}\dot{\Gamma }\left(\Delta \hat{M_{i}}\right)-\sum_{i=1}^{n}\Delta {\hat{f}}_{di}^{i}\circ \left({\hat{W}}_{d}^{-1}\Delta {\dot{\hat{f}}}_{di}^{i}\right) \end{array}$$

(46) $$\begin{array}{cc} {\dot{V}}_{2b}= & \frac{1}{2}{\hat{k}}_{2}⊙\sum_{i=1}^{n}\sum_{j=1}^{n}a_{ij}({\left({\hat{s}}_{j}\right)}^{s}\circ {\left({\hat{s}}_{j}\right)}^{s}\\ \; & -\left(1-{\dot{T}}_{ij}\right){\left({\hat{s}}_{j}\left(t-T_{ij}\right)\right)}^{s}\circ {\left({\hat{s}}_{j}\left(t-T_{ij}\right)\right)}^{s}) \end{array}$$

(47) $$\begin{array}{cc} {\dot{V}}_{2c}= & \sum_{i=1}^{n}\Gamma^{T}\left(\Delta \hat{M_{i}}\right)W_{M}^{-1}\dot{\Gamma }\left(\Delta \hat{M_{i}}\right)+\sum_{i=1}^{n}\Delta {\hat{f}}_{di}^{i}\circ \left({\hat{W}}_{d}^{-1}\Delta {\dot{\hat{f}}}_{di}^{i}\right) \end{array}$$

Then,

(48) $$\begin{array}{cc} {\dot{V}}_{2}= & {\dot{V}}_{a2}+{\dot{V}}_{b2}+{\dot{V}}_{c2}\\ = & \sum_{i=1}^{n}{\left({\hat{s}}_{i}\right)}^{s}\circ {\left(-{\hat{k}}_{1}⊙{\left({\hat{s}}_{i}\right)}^{s}-{\hat{k}}_{2}⊙\sum_{j=1}^{n}a_{ij}\left({\hat{s}}_{i}-{\hat{s}}_{j}(t-T_{ij}\right)\right)}^{s}\\ \; & +\frac{1}{2}{\hat{k}}_{2}⊙\sum_{i=1}^{n}\sum_{j=1}^{n}a_{ij}\left({\left({\hat{s}}_{j}\right)}^{s}\circ {\left({\hat{s}}_{j}\right)}^{s}\right)\\ \; & -\frac{\left(1-{\dot{T}}_{ij}\right)}{2}{\hat{k}}_{2}⊙\sum_{i=1}^{n}\sum_{j=1}^{n}a_{ij}\left({\left({\hat{s}}_{j}\left(t-T_{ij}\right)\right)}^{s}\circ {\left({\hat{s}}_{j}\left(t-T_{ij}\right)\right)}^{s}\right)\\ \; & \leq -{\hat{k}}_{1}⊙\sum_{i=1}^{n}{\left({\hat{s}}_{i}\right)}^{s}\circ {\left({\hat{s}}_{i}\right)}^{s}+{\dot{T}}_{ij}{\hat{k}}_{2}⊙\sum_{i=1}^{n}\sum_{j=1}^{n}a_{ij}\left({\left({\hat{s}}_{j}\left(t-T_{ij}\right)\right)}^{s}\circ {\left({\hat{s}}_{j}\left(t-T_{ij}\right)\right)}^{s}\right) \end{array}$$

Therefore, if ${\dot{T}}_{ij}<0$, it follows that ${\dot{V}}_{2}\left(t\right)\leq 0$. Since $V_{2}\left(t\right)\geq 0$ and ${\dot{V}}_{2}\left(t\right)\leq 0$, ${\hat{s}}_{i}$, $\Gamma ({\tilde{M}}_{i})$, and ${\tilde{f}}_{di}^{i}$ are bounded. Then, based on the similar analysis and proof in the previous section, it can be concluded that $\underset{t\to \infty }{lim}\left({\hat{q}}_{ei},{\hat{\omega }}_{ei}^{i}\right)\left(t\right)=\left(\pm \hat{1},\hat{0}\right)$. We complete the proof. □

**Remark** **1.** *The proposed adaptive law (42) and (43) can only guarantee that $\Delta \hat{M_{i}}$ and $\Delta \hat{f_{di}^{i}}$ are bounded. The estimates of the dual inertia matrix and the external disturbance will not be guaranteed to converge to their actual values.*

**Remark** **2.** *It is worth noting that the dynamic model and controller analysis in this paper is based on continuous time. It is necessary to discretize the controller in the process of practical engineering implementation.*

## 4. Numerical Simulations

This section applies the proposed controller to the earth-centered orbital space gravitational wave detection system. It requires that the variation of the formation arm length (the side length of the triangle) is less than 1%, the relative speed is less than 5 m/s, and the breathing angle (the inner angle of the triangle) is less than $0.1^{\circ }$ [37]. When the position error, velocity error, and attitude error between the actual state and the desired state of the spacecraft should be less than 5 m, 2 mm/s, and 1 mrad, respectively, it can meet the requirements of the gravitational wave detection mission.

The inertia matrix and masses of the spacecraft are assumed to be

(49) $$\begin{array}{cc} J & =\left[\begin{array}{ccc} 162.5 & 3 & 2\\ 3 & 162.5 & 2.5\\ 2 & 2.5 & 325\; \end{array}\right]\mathrm{kg}\cdot m^{2} \end{array}$$

and m=650 kg, respectively. The desired orbit parameters of SC1∼3 are shown in Table 1, assuming that the three spacecraft form an equilateral triangle. The desired attitude and the desired angular velocity are ground orientation and orbital angular velocity, respectively. The initial position errors $r_{ei}={\left[r_{eix},r_{eiy},r_{eiz}\right]}^{T}$, velocity errors ${\dot{r}}_{ei}={\left[{\dot{r}}_{eix},{\dot{r}}_{eiy},{\dot{r}}_{eiz}\right]}^{T}$, angular velocity errors $\omega_{ei}={\left[\omega_{eix},\omega_{eiy},\omega_{eiz}\right]}^{T}$, and attitude errors $\theta_{ei}={\left[\theta_{eix},\theta_{eiy},\theta_{eiz}\right]}^{T}$(i=1,2,3) for each spacecraft are presented in Table 2. Note that we use the Euler angle rather than quaternion to describe the attitude in the simulation, which is easier to understand. The communication time delay between the neighboring spacecraft is supposed to be $T_{ij}=0.6-0.1\times \left|sin\left(0.01t\right)\right|$ s. In this paper, the maximum control forces and torques are set to $10^{-3}$ N and $10^{-4}$N·m in each axis, respectively. The minimum impulse bit is set to $10^{-7}$ N. The position and linear velocity measurement accuracy are assumed to be 0.1 m and $10^{-6}$ m/s, respectively. The attitude and angular velocity measurement accuracy are assumed to be $10^{-6}$ rad and $10^{-7}$ rad/s, respectively. Those measurement errors are assumed to be normally distributed.

### 4.1. 6-DOF Coordinated Control Law with Communication Delays

**Figure 1:**
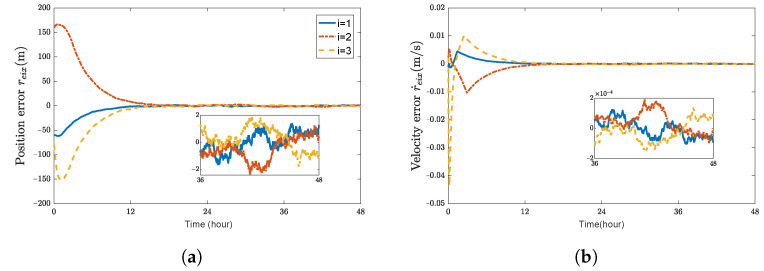

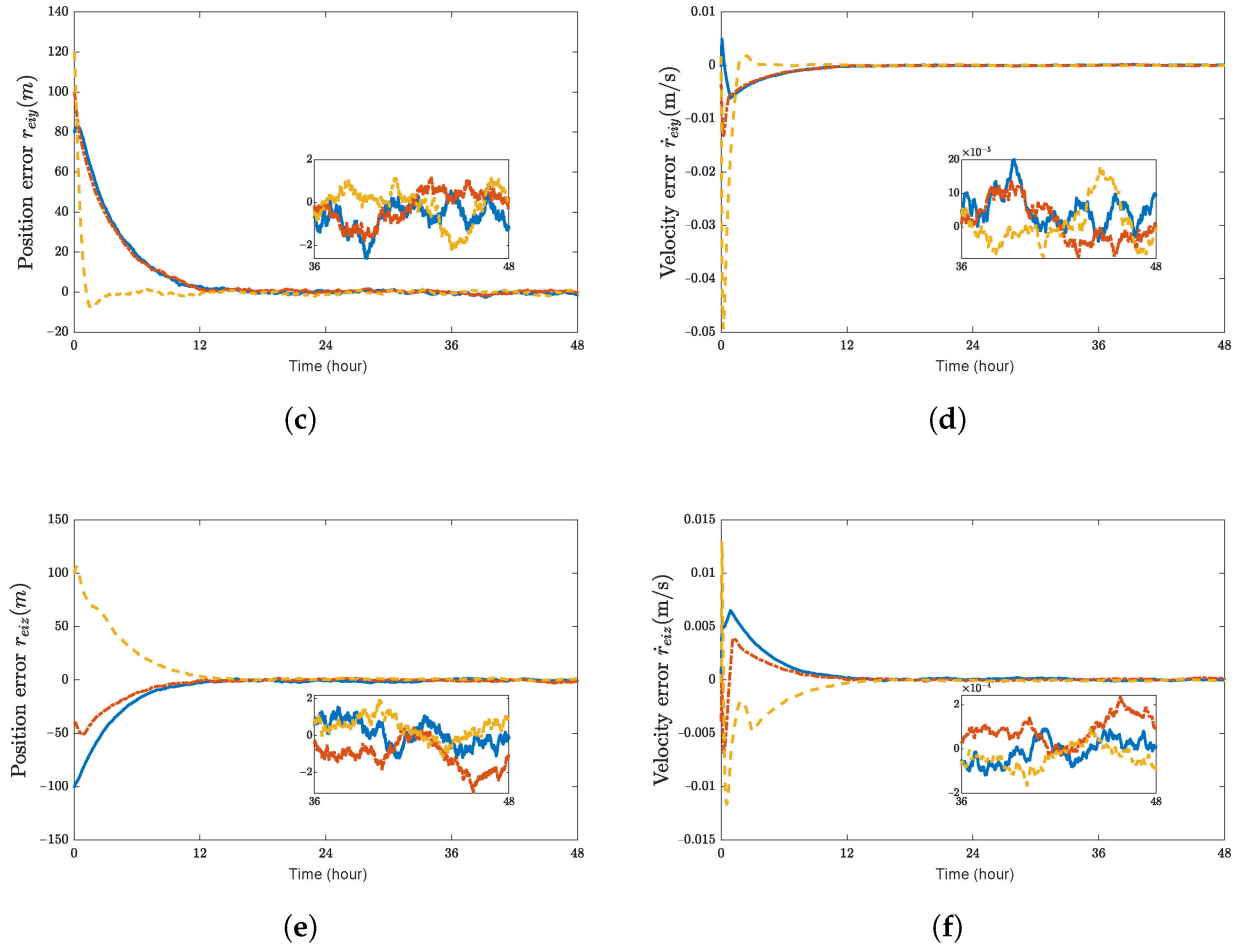
Position errors and velocity errors of spacecraft SC1∼3. (**a**) position error $r_{eix}$ (m); (**b**) velocity error ${\dot{r}}_{eix}$ (m/s); (**c**) position error $r_{eiy}$ (m); (**d**) velocity error ${\dot{r}}_{eiy}$ (m/s); (**e**) position error $r_{eiz}$ (m); (**f**) velocity error ${\dot{r}}_{eiz}$ (m/s).

**Figure 2:**
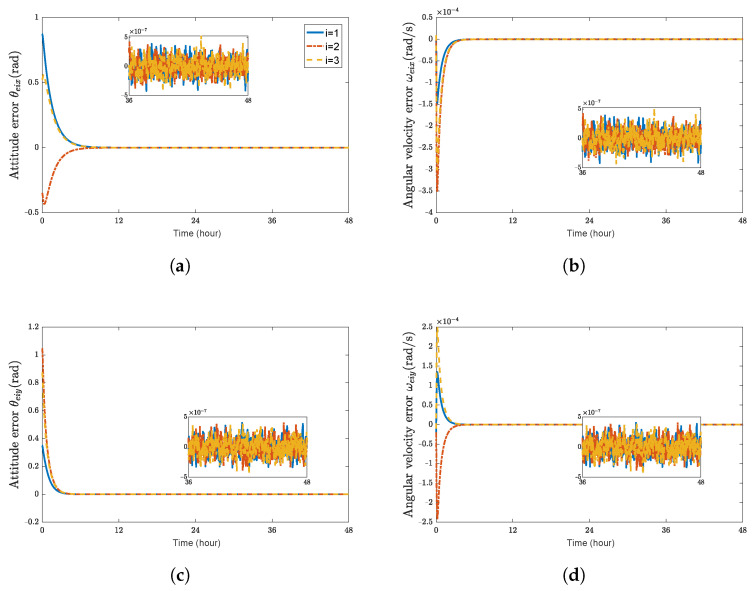

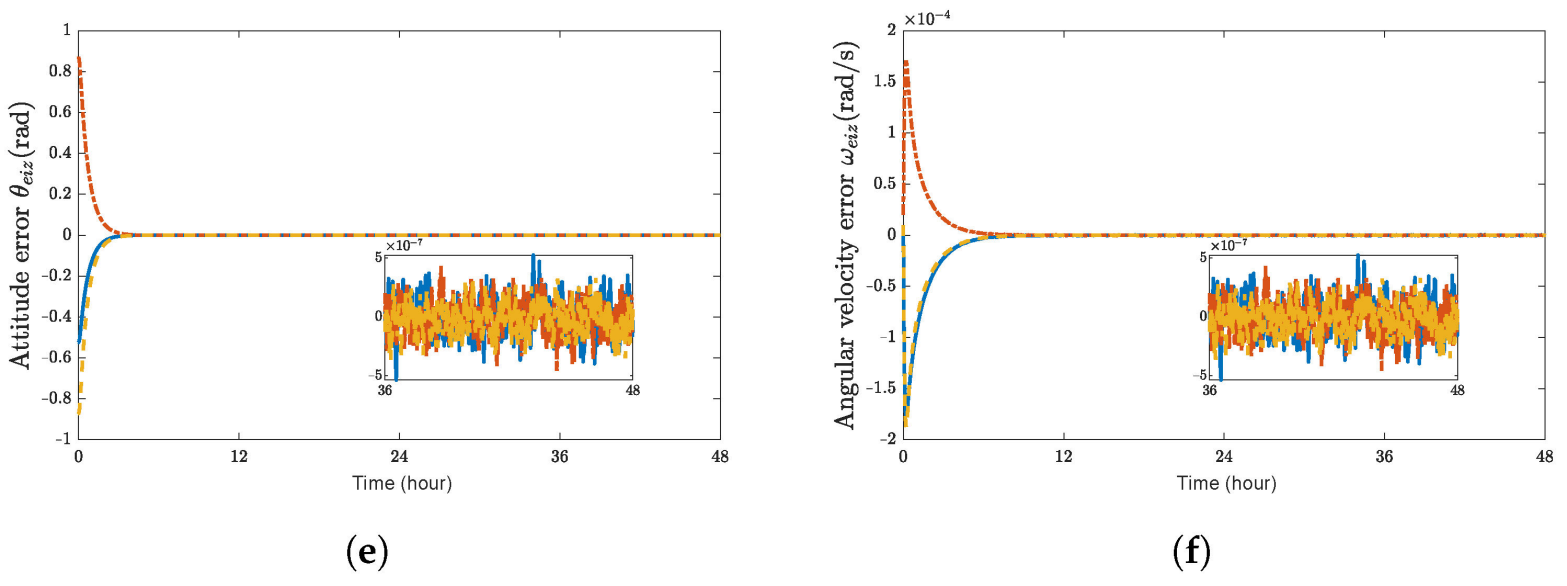
Attitude errors and angular velocity errors of spacecraft SC1∼3. (**a**) attitude error $\theta_{eix}$ (rad); (**b**) angular velocity error $\omega_{eix}$ (rad/s); (**c**) attitude error $\theta_{eiy}$ (rad); (**d**) angular velocity error $\omega_{eiy}$ (rad/s); (**e**) attitude error $\theta_{eiz}$ (rad); (**f**) angular velocity error $\omega_{eiz}$ (rad/s).

**Figure 3:**
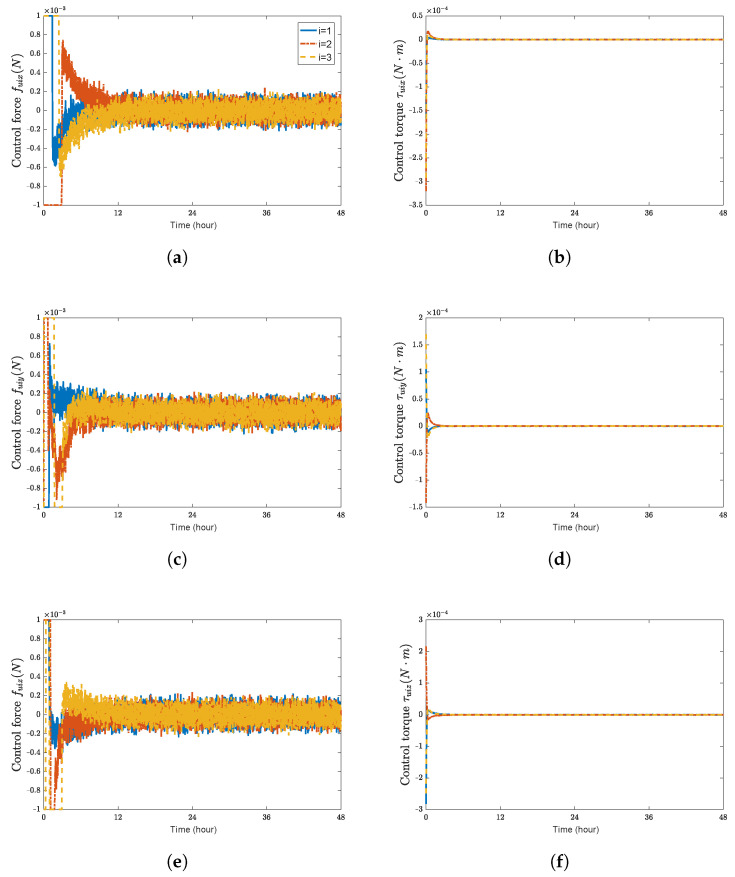
Control forces and control torques of spacecraft SC1∼3. (**a**) control force $f_{uix}\left(N\right)$; (**b**) control torque $\tau_{uix}\left(N\cdot m\right)$; (**c**) control force $f_{uiy}\left(N\right)$; (**d**) control torque $\tau_{uiy}\left(N\cdot m\right)$; (**e**) control force $f_{uiz}\left(N\right)$; (**f**) control torque $\tau_{uiz}\left(N\cdot m\right)$.

Using a trial and error procedure, we select the gains for the controller (29) as $k_{1d}=0.05$, $k_{1r}=0.06$, $k_{2d}=0.001$, $k_{2r}=0.001$, $c_{r}=0.01$, and $c_{d}=0.035$. Under the designed 6-DOF coordination control law (29), simulation results are presented in Figure 1, Figure 2 and Figure 3, which validate the stability analysis of the proposed control schemes.

Figure 1 shows the time histories of the position errors and linear velocity errors of each spacecraft with communication delays, respectively. It can be seen that the position errors and linear velocity errors converge to the region $|r_{eiw}|<2.5$ m and $|{\dot{r}}_{eiw}|<2\times 10^{-4}$ m/s, (w=x,y,z). The performance of position tracking and linear velocity tracking during the transient phase and the final accuracy is acceptable. Figure 2 shows the time histories of attitude errors and angular velocity errors of each spacecraft with communication delays, respectively. It can be observed that attitude errors and angular velocity errors converge to the region $|\theta_{eiw}|<5\times 10^{-4}$ rad and $|\omega_{eiw}|<5\times 10^{-7}$ rad/s. Figure 3 shows the control forces and control torques of each spacecraft.

As shown in Figure 1 and Figure 2, it can be seen that the convergence time is about 18 h and 12 h for the translation and rotation, respectively. These two figures indicate that the errors of the relative position and attitude could rapidly converge and satisfy the accuracy requirements when the time-varying communication delay is considered.

### 4.2. Adaptive 6-DOF Coordinated Control Law with Communication Delays

**Figure 4:**
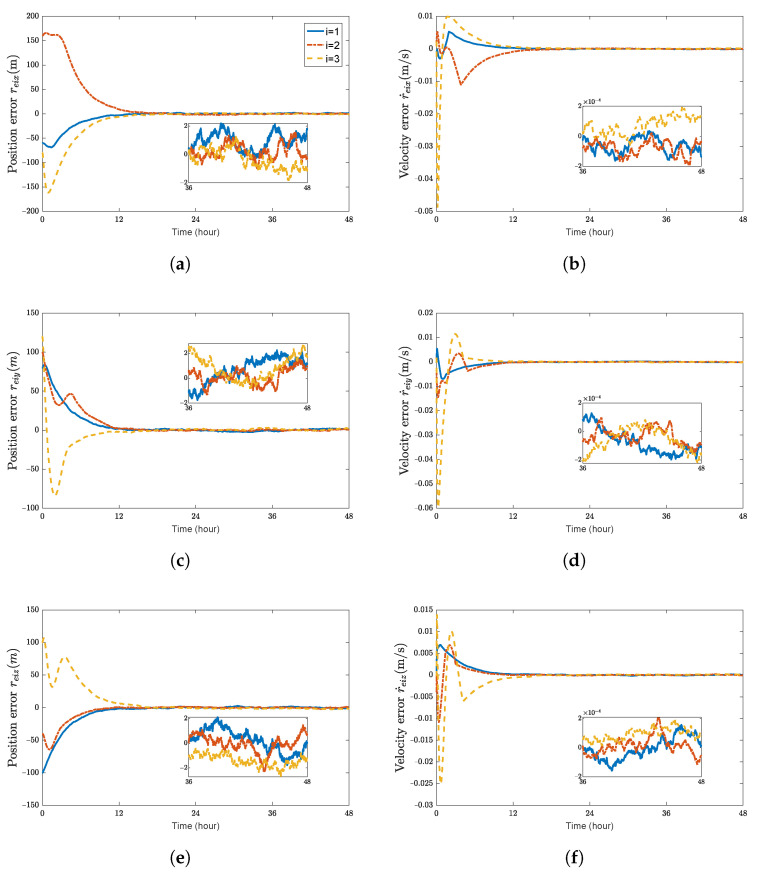
Position errors and velocity errors of spacecraft SC1∼3 with adaptive control law. (**a**) position error $r_{eix}$ (m); (**b**) velocity error ${\dot{r}}_{eix}$ (m/s); (**c**) position error $r_{eiy}$ (m); (**d**) velocity error ${\dot{r}}_{eiy}$ (m/s); (**e**) position error $r_{eiz}$ (m); (**f**) velocity error ${\dot{r}}_{eiz}$ (m/s).

**Figure 5:**
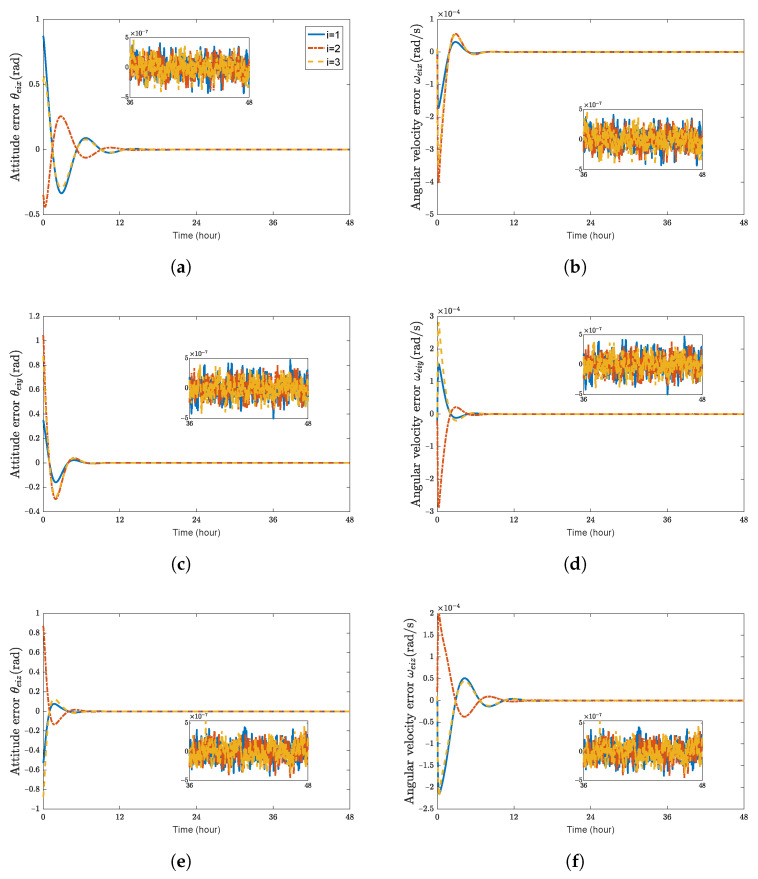
Attitude errors and angular velocity errors of spacecraft SC1∼3 with adaptive control law. (**a**) attitude error $\theta_{eix}$ (rad); (**b**) angular velocity error $\omega_{eix}$ (rad/s); (**c**) attitude error $\theta_{eiy}$ (rad); (**d**) angular velocity error $\omega_{eiy}$ (rad/s); (**e**) attitude error $\theta_{eiz}$ (rad); (**f**) angular velocity error $\omega_{eiz}$ (rad/s).

**Figure 6:**
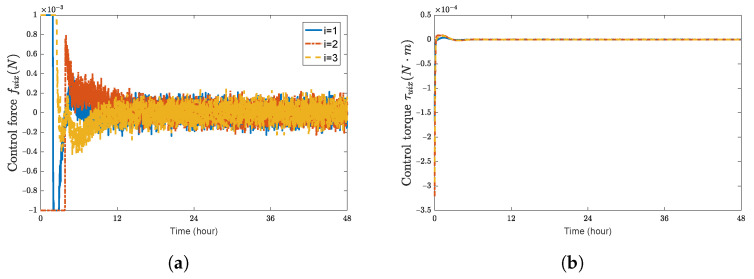

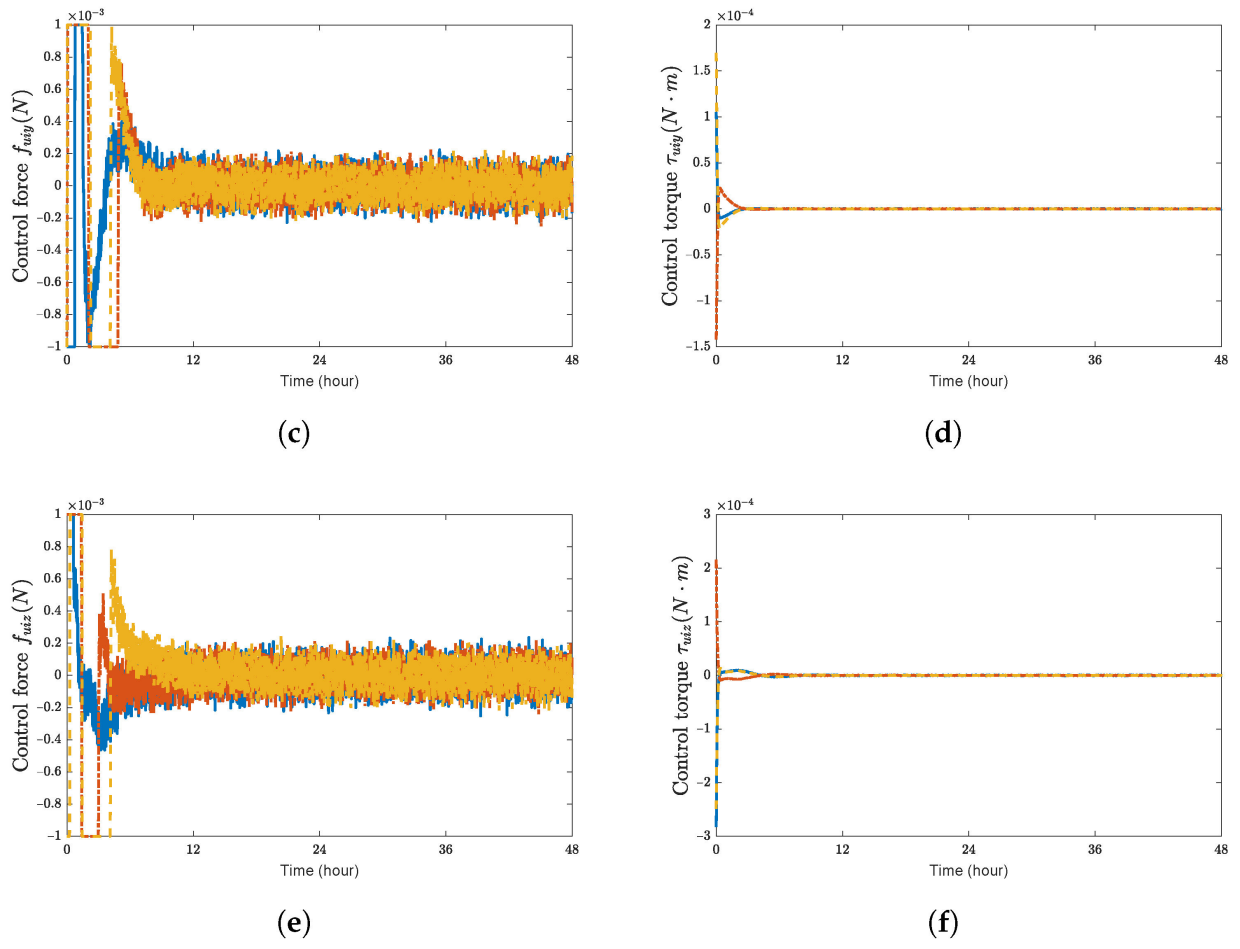
Control forces and control torques of spacecraft SC1∼3 with adaptive control law. (**a**) control force $f_{uix}\left(N\right)$; (**b**) control torque $\tau_{uix}\left(N\cdot m\right)$; (**c**) control force $f_{uiy}\left(N\right)$; (**d**) control torque $\tau_{uiy}\left(N\cdot m\right)$; (**e**) control force $f_{uiz}\left(N\right)$; (**f**) control torque $\tau_{uiz}\left(N\cdot m\right)$.

**Figure 7:**
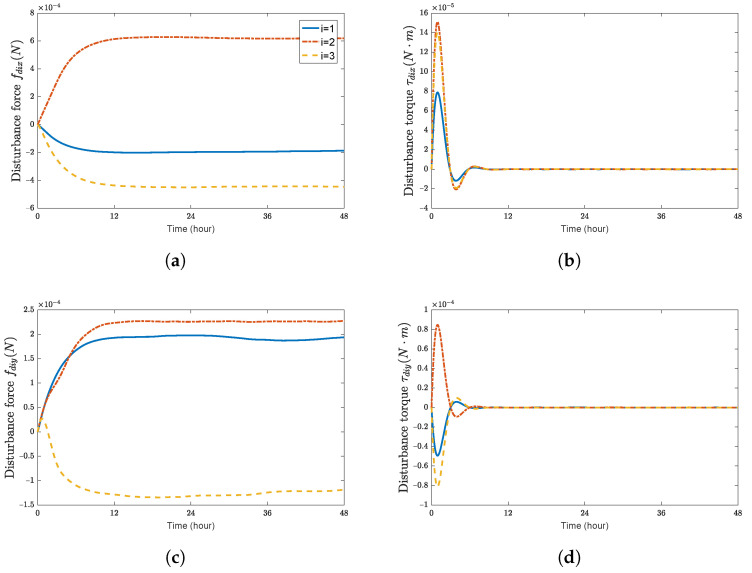

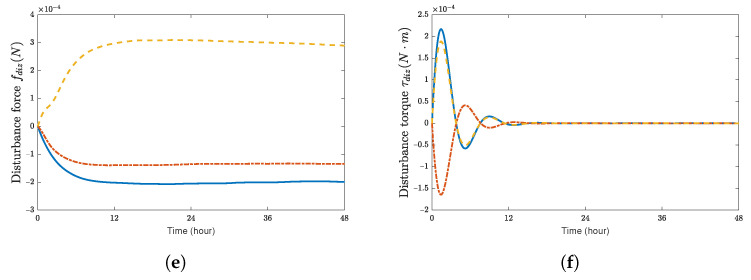
Disturbance forces and disturbance torques of spacecraft SC1∼3 with adaptive control law. (**a**) disturbance force $f_{dix}\left(N\right)$; (**b**) disturbance torque $\tau_{dix}\left(N\cdot m\right)$; (**c**) disturbance force $f_{diy}\left(N\right)$; (**d**) disturbance torque $\tau_{diy}\left(N\cdot m\right)$; (**e**) disturbance force $f_{diz}\left(N\right)$; (**f**) disturbance torque $\tau_{diz}\left(N\cdot m\right)$.

**Figure 8:**
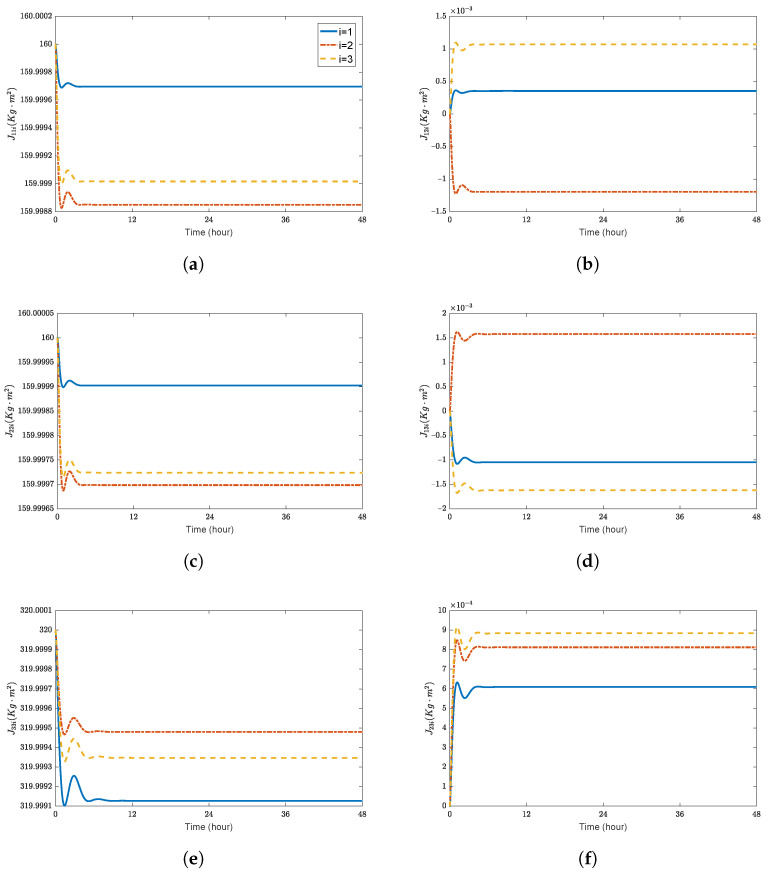

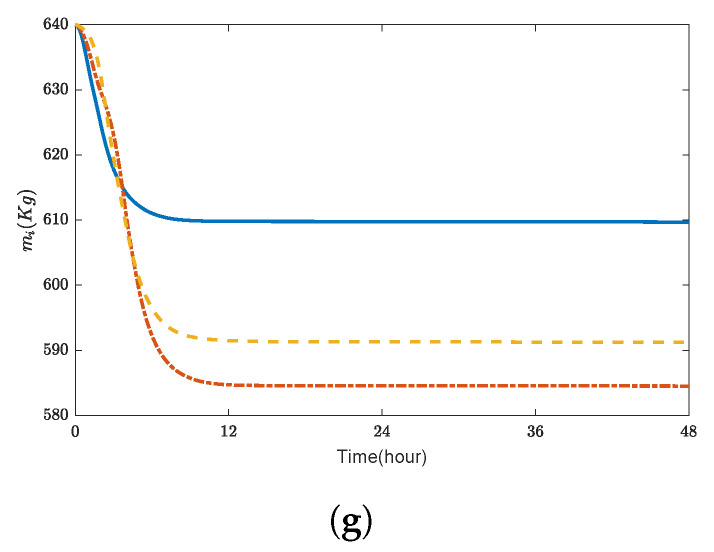
Estimation of the inertia matrix and mass under the proposed adaptive controller. (**a**) $J_{11i}\left(\mathrm{Kg}\cdot m^{2}\right)$; (**b**) $J_{12i}\left(\mathrm{Kg}\cdot m^{2}\right)$; (**c**) $J_{22i}\left(\mathrm{Kg}\cdot m^{2}\right)$; (**d**) $J_{13i}\left(\mathrm{Kg}\cdot m^{2}\right)$; (**e**) $J_{33i}\left(\mathrm{Kg}\cdot m^{2}\right)$; (**f**) $J_{23i}\left(\mathrm{Kg}\cdot m^{2}\right)$; (**g**) $m_{i}\left(\mathrm{Kg}\right)$.

Considering the 6-DOF coordination adaptive control law (39), (40), (42), and (43), selecting the adaptive gain parameter as $W_{M}=10\times diag\left\{1,1,1,1,1,1,1\right\}$, $W_{d1}=10^{-5}I_{3}$, $W_{d2}=2\times 10^{-2}I_{3}$. The initial values of the estimated variables are set to ${\tilde{f}}_{di}^{i}\left(0\right)=\hat{0}$ and $\Gamma \left({\hat{M}}_{i}\right)\left(0\right)={\left[160,0,0,160,0,320,640\right]}^{T}$. Other parameters for control law (39) remain the same as those in the previous section. The simulation figures are given in Figure 4, Figure 5, Figure 6, Figure 7 and Figure 8.

Figure 4 and Figure 5 show the time histories of the position errors, linear velocity errors, attitude errors, and angular velocity errors of each spacecraft with communication delays, model uncertainties, and external disturbances, respectively. It can be seen that the spacecraft can asymptotically track their desired positions, and the tracking error can converge to the region $|r_{eiw}|<2.5$ m, $|{\dot{r}}_{eiw}|<2\times 10^{-4}$ m/s, $|\theta_{eiw}|<3\times 10^{-4}$ rad, and $|\omega_{eiw}|<5\times 10^{-7}$ rad/s, (w=x,y,z).

Figure 6 shows the control forces and control torques of each spacecraft, respectively. The estimation of the external disturbances, the inertia matrix, and the mass under the proposed adaptive controller are shown in Figure 7 and Figure 8. Although the updating laws given by (42) and (43) do not converge to the actual values of the spacecraft, the asymptotic convergence of the position errors and the attitude errors are still guaranteed.

The transient response of the control law (39) is less smooth than that of the control law (29), which does not consider parametric uncertainties and external interference. This is because it takes time for the updating law (42) and (43) to adjust the estimations of the dual inertia and external disturbances to achieve a fine compensation. However, the settling time for the two controllers is identical, and the accuracy of the relative position errors and attitude errors are the same, which can meet the requirements of gravitational wave detection for the initial pose error.

## 5. Conclusions

This paper has investigated the attitude and orbit coupled tracking control problem for multiple spacecraft formation. A distributed 6-DOF coordinated control law based on dual quaternions has been designed with time-varying communication delays. Moreover, an adaptive control law has been further developed by consideration of parametric uncertainties and external disturbances, where the asymptotic stability of the closed-loop system is guaranteed. Numerical simulation results show that the controller can realize the coordination of relative orbit and attitude, and make the formation configuration meet the requirements of space gravitational wave detection. In future work, the distributed attitude-orbit coordinated control with velocity-free could be studied.

## Figures and Tables

**Table 1:** Desired orbital parameters.

Parameter	Value	Unit
Perigee altitude	$9.999\times 10^{7}$	m
Eccentricity	0.00043	-
Inclination	74.5362	deg
Argument of perigee	346.5528	deg
RAAN	211.6003	deg
True anomaly (SC1)	61.3296	deg
True anomaly (SC2)	181.3296	deg
True anomaly (SC3)	301.3296	deg

**Table 2:** Initial conditions.

	Initial Position Error (m)	Initial Velocity Error ($m\cdot s^{-1}$)	Initial Angular Velocity Error ($\mathrm{rad}\cdot s^{-1}$)	Initial Attitude Error (rad)
SC1	[−60 80 −100]${}^{T}$	[1.2 −0.22 0.57]${}^{T}\times 10^{-3}$	[0.8 −2 1] ${}^{T}\times 10^{-5}$	[0.8727 −0.5236 0.3491]
SC2	[160 100 −40]${}^{T}$	[1.2 −3.5 −3.9] ${}^{T}\times 10^{-3}$	[0.7 −2 2] ${}^{T}\times 10^{-5}$	[−0.3491 0.8727 1.0472]
SC3	[−80 120 100]${}^{T}$	[2.2 1.7 −0.29] ${}^{T}\times 10^{-3}$	[0.9 −1 1] ${}^{T}\times 10^{-5}$	[0.5236 −0.8727 0.8727]

## Data Availability

Not applicable.

## References

[1] Abbott B.P., Abbott R., Abbott T., Abernathy M., Acernese F., Ackley K., Adams C., Adams T., Addesso P., Adhikari R. (2016). Observation of gravitational waves from a binary black hole merger. Phys. Rev. Lett..

[2] Stebbins R.T. (2009). Rightsizing LISA. Class. Quantum Gravity.

[3] Luo J., Chen L.S., Duan H.Z., Gong Y.G., Hu S., Ji J., Liu Q., Mei J., Milyukov V., Sazhin M. (2016). TianQin: A space-borne gravitational wave detector. Class. Quantum Gravity.

[4] Hu W.R., Wu Y.L. (2017). Taiji program in space for gravitational wave physics and nature of gravity. Natl. Sci. Rev..

[5] Zhang F., Duan G. (2013). Robust adaptive integrated translation and rotation control of a rigid spacecraft with control saturation and actuator misalignment. Acta Astronaut..

[6] Ye D., Zhang J., Sun Z. (2017). Extended state observer–based finite-time controller design for coupled spacecraft formation with actuator saturation. Adv. Mech. Eng..

[7] Nazari M., Butcher E.A., Yucelen T., Sanyal A.K. (2016). Decentralized consensus control of a rigid-body spacecraft formation with communication delay. J. Guid. Control. Dyn..

[8] Zhang J., Ye D., Liu M., Sun Z. (2017). Adaptive fuzzy finite-time control for spacecraft formation with communication delays and changing topologies. J. Frankl. Inst..

[9] Liu R., Cao X., Liu M., Zhu Y. (2019). 6-DOF fixed-time adaptive tracking control for spacecraft formation flying with input quantization. Inf. Sci..

[10] Filipe N., Tsiotras P. (2015). Adaptive position and attitude-tracking controller for satellite proximity operations using dual quaternions. J. Guid. Control. Dyn..

[11] Gui H., Vukovich G. (2016). Dual-quaternion-based adaptive motion tracking of spacecraft with reduced control effort. Nonlinear Dyn..

[12] Adorno B.V. (2011). Two-Arm Manipulation: From Manipulators to Enhanced Human-Robot Collaboration. Ph.D. Thesis.

[13] Wang Y., Yuan Y., Liu J. (2021). Finite-time leader-following output consensus for multi-agent systems via extended state observer. Automatica.

[14] Lu M., Liu L. (2019). Leader-following attitude consensus of multiple rigid spacecraft systems under switching networks. IEEE Trans. Autom. Control.

[15] Mesbahi M., Hadaegh F.Y. Formation flying control of multiple spacecraft via graphs, matrix inequalities, and switching. Proceedings of the 1999 IEEE International Conference on Control Applications (Cat. No. 99CH36328).

[16] Zhang Y., Yang L., Zhu Y., Huang H., Cai W. (2012). Nonlinear 6-DOF control of spacecraft docking with inter-satellite electromagnetic force. Acta Astronaut..

[17] PARI H.M., Bolandi H. (2021). Discrete time multiple spacecraft formation flying attitude optimal control in the presence of relative state constraints. Chin. J. Aeronaut..

[18] Bennet D.J., McInnes C.R. (2012). Pattern transition in spacecraft formation flying using bifurcating potential fields. Aerosp. Sci. Technol..

[19] Hong H., Yu C., Yu W. (2022). Adaptive fixed-time control for attitude consensus of disturbed multi-spacecraft systems with directed topologies. IEEE Trans. Netw. Sci. Eng..

[20] Shahbazi B., Malekzadeh M., Koofigar H.R. (2017). Robust constrained attitude control of spacecraft formation flying in the presence of disturbances. IEEE Trans. Aerosp. Electron. Syst..

[21] De Queiroz M.S., Kapila V., Yan Q. (2000). Adaptive nonlinear control of multiple spacecraft formation flying. J. Guid. Control. Dyn..

[22] Lee K.W., Singh S.N. (2012). Variable-structure model reference adaptive formation control of spacecraft. J. Guid. Control. Dyn..

[23] Sankaranarayanan V.N., Satpute S., Nikolakopoulos G. (2022). Adaptive Robust Control for Quadrotors with Unknown Time-Varying Delays and Uncertainties in Dynamics. Drones.

[24] Wang H., Ma K., Wu S., Li M., Lian X., Zhang J. (2023). Robust tracking control of unknown models for space in-cabin robots with a pneumatic continuum arm. Complex Intell. Syst..

[25] Zhang C., Wang J., Zhang D., Shao X. (2018). Fault-tolerant adaptive finite-time attitude synchronization and tracking control for multi-spacecraft formation. Aerosp. Sci. Technol..

[26] Xing L., Zhang J., Liu C., Zhang X. (2021). Fuzzy-logic-based adaptive event-triggered sliding mode control for spacecraft attitude tracking. Aerosp. Sci. Technol..

[27] Lin X., Shi X., Li S. (2020). Adaptive tracking control for spacecraft formation flying system via modified fast integral terminal sliding mode surface. IEEE Access.

[28] Zhu X., Chen J., Zhu Z.H. (2021). Adaptive sliding mode disturbance observer-based control for rendezvous with non-cooperative spacecraft. Acta Astronaut..

[29] Yang J., Stoll E. (2019). Adaptive sliding mode control for spacecraft proximity operations based on dual quaternions. J. Guid. Control. Dyn..

[30] Wu J., Liu K., Han D. (2013). Adaptive sliding mode control for six-DOF relative motion of spacecraft with input constraint. Acta Astronaut..

[31] Huang Y., Jia Y. (2019). Adaptive finite time distributed 6-DOF synchronization control for spacecraft formation without velocity measurement. Nonlinear Dyn..

[32] Zhang J., Qinglei H., Danwei W., Wenbo X. (2017). Robust attitude coordinated control for spacecraft formation with communication delays. Chin. J. Aeronaut..

[33] Yuan Y., Wang Y., Guo L. (2020). Sliding-mode-observer-based time-varying formation tracking for multispacecrafts subjected to switching topologies and time-delays. IEEE Trans. Autom. Control.

[34] Zhang J., Hu Q., Xie W. (2017). Integral sliding mode-based attitude coordinated tracking for spacecraft formation with communication delays. Int. J. Syst. Sci..

[35] Brodsky V., Shoham M. (1999). Dual numbers representation of rigid body dynamics. Mech. Mach. Theory.

[36] Wang J., Sun Z. (2012). 6-DOF robust adaptive terminal sliding mode control for spacecraft formation flying. Acta Astronaut..

[37] Ye B.B., Zhang X., Zhou M.Y., Wang Y., Yuan H.M., Gu D., Ding Y., Zhang J., Mei J., Luo J. (2019). Optimizing orbits for TianQin. Int. J. Mod. Phys. D.

